# Amino Acid-Derived Metabolic Signature Across Stages of Systolic Dysfunction: Derivation and Internal Evaluation of the HASI (Heart Failure Amino Acid-Derived Systolic Index)—40 Index

**DOI:** 10.3390/ijms27104459

**Published:** 2026-05-15

**Authors:** Beata Krasińska, Ievgen Spasenenko, Dagmara Pietkiewicz, Szymon Plewa, Krzysztof J. Filipiak, Katarzyna Pawlaczyk-Gabriel, Jarosław Bartkowski, Andrzej Tykarski, Zbigniew Krasiński, Jan Matysiak, Tomasz Urbanowicz

**Affiliations:** 1 Department of Hypertensiology, Angiology, and Internal Medicine, Poznan University of Medical Sciences, 1/2 Długa Street, 61-848 Poznań, Poland; 2 Department of Inorganic and Analytical Chemistry, Faculty of Pharmacy, Poznan University of Medical Sciences, 1/2 Długa Street, 61-848 Poznań, Poland; 3 The Centre of Postgraduate Medical Education, 99/103 Marymoncka Street, 01-813 Warsaw, Poland; 4 Cardiac Surgery and Transplantology Department, Poznan University of Medical Sciences, 1/2 Długa Street, 61-848 Poznan, Poland; 5 Department of Vascular, Endovascular Surgery, Angiology and Phlebology, Poznan University of Medical Science, 1/2 Dluga Street, 61-848 Poznan, Poland

**Keywords:** HFrEF, HFmrEF, amino acids, metabolomics

## Abstract

Heart failure with reduced ejection fraction (HFrEF) is increasingly recognized as a systemic metabolic disorder. The aim of this study was to characterize amino acid-related metabolic differences between heart failure with moderately reduced ejection fraction (HFmrEF) (LVEF 40–49%) and HFrEF (LVEF < 40%) and to derive a biologically interpretable composite metabolomic index capable of discriminating between these two stages of systolic dysfunction. We conducted a cross-sectional metabolomic analysis of 42 patients stratified by left ventricular ejection fraction (LVEF < 40% vs. 40–49%). The reference group comprised patients with mildly reduced ejection fraction (LVEF 40–49%), without inclusion of individuals with preserved or normal cardiac function. Targeted amino acid profiling was performed using liquid chromatography-tandem mass spectrometry (LC–MS/MS). Metabolites were standardized and analyzed individually and in combination. A composite index (Heart Failure Amino Acid-Derived Systolic Index: HASI-40), integrating markers of proteolysis and metabolic resilience, was derived to distinguish patients with HFrEF from those with HFmrEF. Discrimination was assessed using receiver operator curve (ROC) analysis with internal validation and multivariable adjustment. Patients with LVEF < 40% exhibited a coordinated metabolic phenotype characterized by reduced methionine, sarcosine, serine, and taurine. While individual metabolites did not retain significance after multiple-testing correction, the composite HASI-40 index remained strongly associated with HFrEF (OR 5.56, 95% CI: 1.70–18.14; *p* = 0.004), although the wide confidence interval indicates limited precision due to sample size. The index demonstrated good discrimination with an area under the curve (AUC) of 0.862, which improved when combined with age (AUC 0.932). The index represents a standardized composite measure and does not define a diagnostic cutoff for individual patients. These findings suggest that HFmrEF and HFrEF exhibit partially distinct metabolic phenotypes despite overlapping clinical characteristics. These findings suggest that HASI-40 captures metabolic differences between patients with HFmrEF (LVEF 40–49%) and those with HFrEF (LVEF < 40%), reflecting progression toward more advanced systolic dysfunction. However, due to the absence of a control group with preserved ejection fraction, small sample size, and lack of external validation, the index should be considered exploratory and hypothesis-generating rather than clinically applicable.

## 1. Introduction

Heart failure with reduced ejection fraction (HFrEF) represents the final common pathway of diverse cardiovascular insults, yet its pathophysiology extends beyond impaired myocardial contractility [1,2]. Increasing evidence supports the view that HFrEF is a systemic disorder characterized by profound disturbances in energy metabolism, substrate utilization, and inter-organ metabolic communication [3,4,5]. These alterations involve coordinated changes across skeletal muscle, liver, and myocardium, reflecting a loss of metabolic flexibility rather than isolated organ dysfunction [6,7,8].

Among circulating metabolites, amino acids occupy a central regulatory position at the interface of nitrogen balance, redox homeostasis, and mitochondrial energetics [9,10,11]. In the setting of heart failure, amino acid metabolism is shaped by competing processes: activation of proteolysis, impaired anabolic signaling, and disruption of one-carbon metabolism. These pathways are not independent. Instead, they converge on shared biological functions, including mitochondrial function, oxidative stress regulation, and cellular repair. Despite this, prior metabolomic studies in heart failure have largely focused on individual metabolites or pathway-specific alterations, resulting in heterogeneous findings with limited clinical applicability [12,13,14].

Previous studies [15,16,17] have demonstrated alterations in amino acids such as taurine, methionine, and serine in heart failure across larger cohorts, supporting their roles in mitochondrial function, redox balance, and metabolic regulation. However, these findings have been heterogeneous and difficult to translate into clinically interpretable markers.

Heart failure with mildly reduced ejection fraction (HFmrEF) occupies an intermediate position within the heart failure spectrum, yet its biological identity remains incompletely defined [18,19]. While HFrEF is characterized by extensive structural remodeling and dominant systolic dysfunction, HFmrEF appears to retain elements of both systolic and diastolic impairment, often in the context of a distinct comorbidity profile [20]. Importantly, whether HFmrEF represents a transitional stage or a separate pathophysiological entity remains debated [21,22]. From a metabolic perspective, this distinction is critical, as it may reflect differences in the integrity of adaptive metabolic pathways rather than solely differences in ejection fraction.

We theorized that a composite amino acid-derived signature integrating key domains of metabolic regulation would better capture the systemic metabolic state associated with systolic dysfunction than individual metabolites alone. Specifically, we focused on three biologically interconnected axes: proteolytic activation, reflecting systemic catabolic drive; cytoprotective capacity, representing the ability to buffer oxidative and metabolic stress; and methylation-dependent one-carbon metabolism, which governs mitochondrial function, redox balance, and cellular repair processes. The integration of these domains provides a framework for conceptualizing heart failure as a disorder of coordinated metabolic network failure.

**Figure 1:**
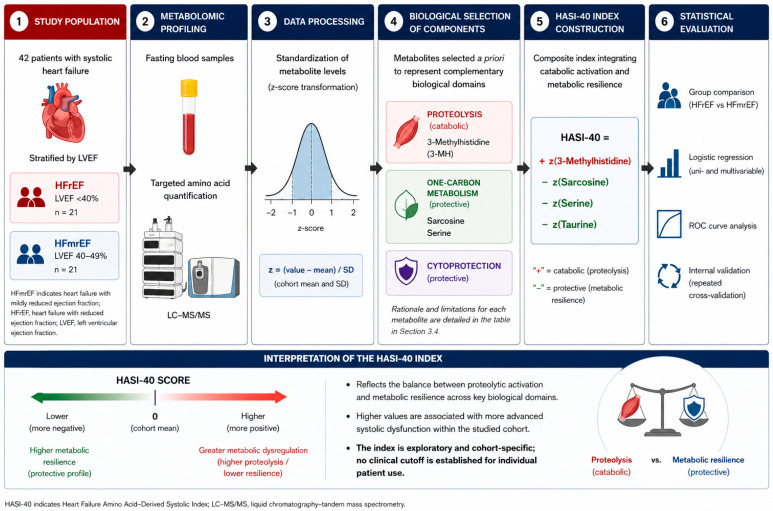
Workflow for derivation of the HASI-40 index. The figure illustrates the stepwise process of index development, including patient stratification, targeted metabolomic profiling, data standardization using z-scores, biologically guided selection of metabolites representing distinct domains (proteolysis, one-carbon metabolism, and cytoprotection), construction of the composite index, and its statistical evaluation. The HASI-40 index reflects the balance between proteolytic activation and metabolic resilience and should be interpreted as an exploratory, cohort-specific measure without a clinically validated cutoff.

To operationalize this concept, we developed the Heart Failure Amino Acid-Derived Systolic Index-40 (HASI-40 index), a composite biomarker derived from circulating amino acids that reflects these complementary biological processes (Figure 1).

We postulated that HASI-40 would discriminate patients with HFrEF from those with HFmrEF and capture a multidimensional metabolic phenotype associated with systolic dysfunction. Accordingly, the aims of the present study were threefold: (1) to characterize the amino acid profile associated with LVEF < 40%, (2) to derive a biologically grounded composite index integrating key metabolic axes, and (3) to evaluate its discriminatory performance and clinical relevance in comparison with conventional clinical parameters.

The selection of HFmrEF (LVEF 40–49%) as the reference group was intended to provide a clinically relevant comparison within the spectrum of systolic dysfunction rather than against healthy individuals. This approach allows exploration of metabolic transitions between partially preserved and overtly impaired systolic function, although it does not permit conclusions regarding normal physiology.

We hypothesized that a composite metabolomic index integrating biologically distinct domains—proteolysis, cytoprotective buffering, and one-carbon metabolism—would capture systemic metabolic dysregulation associated with more advanced systolic impairment within a heart failure population.

The choice of HFmrEF as a reference group was not only pragmatic but conceptually aligned with the study objective, which was to capture metabolic transitions within the heart failure spectrum rather than differences between health and disease. This design prioritizes the identification of progression-related metabolic shifts, although it limits conclusions regarding disease specificity.

The aim of the present study was to determine whether circulating amino acid profiles can distinguish patients with HFmrEF (LVEF 40–49%) from those with HFrEF (LVEF < 40%) and to derive a biologically interpretable composite metabolomic index reflecting progression of systolic dysfunction. We hypothesized that integration of metabolites related to proteolysis, one-carbon metabolism, and cytoprotective buffering would better capture systemic metabolic dysregulation associated with advanced systolic impairment than individual metabolites alone.

## 2. Results and Discussion

### 2.1. Groups Characterization

There were 42 patients enrolled in the amino acids study, divided into equal-sized groups based on left ventricular ejection fraction (LVEF): 40–49% and LVEF < 40%, respectively. There were no differences in demographics or comorbidities between the groups, as presented in Table 1, except for clinical status.

Importantly, the groups differed significantly in NYHA functional class, with more advanced symptoms in the LVEF < 40% group. This introduces potential confounding, as metabolic alterations may reflect disease severity rather than systolic dysfunction per se. This limitation was not directly adjusted for in multivariable models and should be considered when interpreting the findings.

### 2.2. Laboratory Results

The laboratory test performed on admission did not point out significant differences between the analyzed groups, including brain natriuretic peptide (BNP) serum concentration (*p* = 0.221), as shown in Table 2.

Although BNP concentrations were numerically higher in the LVEF 40–49% group, this difference was not statistically significant and may reflect heterogeneity in volume status, diastolic dysfunction, or sampling variability. However, the possibility of residual clinical heterogeneity or misclassification cannot be fully excluded and should be interpreted with caution.

The echocardiography results indicated significant differences in left ventricular diastolic diameter (*p* = 0.018) and left ventricular ejection fraction (*p* < 0.001).

### 2.3. Metabolomic Profiling

Serum amino acid concentrations in the analyzed group were investigated in relation to their metabolic roles, including proteinogenic, one-carbon, and muscle metabolism (Table 3).

The metabolomic analysis revealed a structured, biologically coherent pattern rather than isolated metabolite alterations. Although several amino acids differed nominally between groups, none remained statistically significant after false discovery rate correction, underscoring the limitation of single-metabolite approaches in capturing systemic metabolic states. Significantly lower circulating methionine (*p* = 0.035), tyrosine (*p* = 0.032), sarcosine (*p* = 0.004), serine (*p* = 0.012), and taurine (*p* = 0.018) concentrations were observed in the HFrEF group. After Benjamini–Hochberg false discovery rate correction across the amino acid panel, these individual differences no longer remained significant, with sarcosine showing the strongest residual signal.

### 2.4. HASI-40 Index

**Figure 2:**
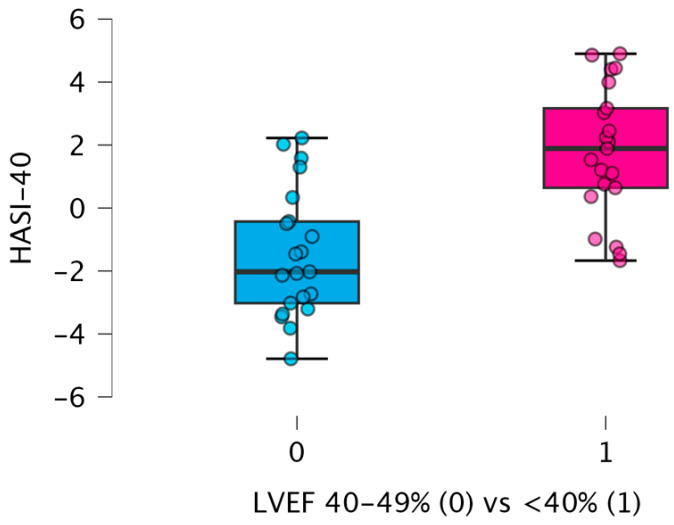
Differences in HASI-40 index between patients with LVEF 40–49% (0) and LVEF < 40% (1).

In contrast, the composite HASI-40 index, integrating markers of proteolysis (3-methylhistidine) and metabolic resilience (sarcosine, serine, taurine), demonstrated a robust and statistically stable association with HFrEF. The persistence of significance after multiple-testing correction suggests that the metabolic phenotype of systolic dysfunction is best represented at the integrative level rather than by individual analytes. The HASI-40 index comparison between the analyzed groups (HFrEF vs. HFmrEF) showed significant differences *p* < 0.001). The HASI-40 index remained strongly associated with HFrEF (*p* < 0.001) and, unlike individual metabolites, its significance was preserved under multiple-testing considerations, as shown in Figure 2.

Although values are visually separated around zero, this threshold is cohort-derived and should not be used clinically. Our results of metabolic phenotyping in HFrEF (LVEF < 40%) exhibited a coordinated metabolic shift based on increased proteolysis (increased circulating 3-methylhistidine), accompanied by decreased cytoprotection (decreased taurine concentration), diminished one-carbon metabolism (lower circulating serine), and methylation flux (described by lower sarcosine concentrations). This pattern reflects systemic metabolic decompensation rather than isolated pathway disruption.

### 2.5. Correlations Between Circulating Amino Acids and Left Ventricular Ejection Fraction

**Figure 3:**
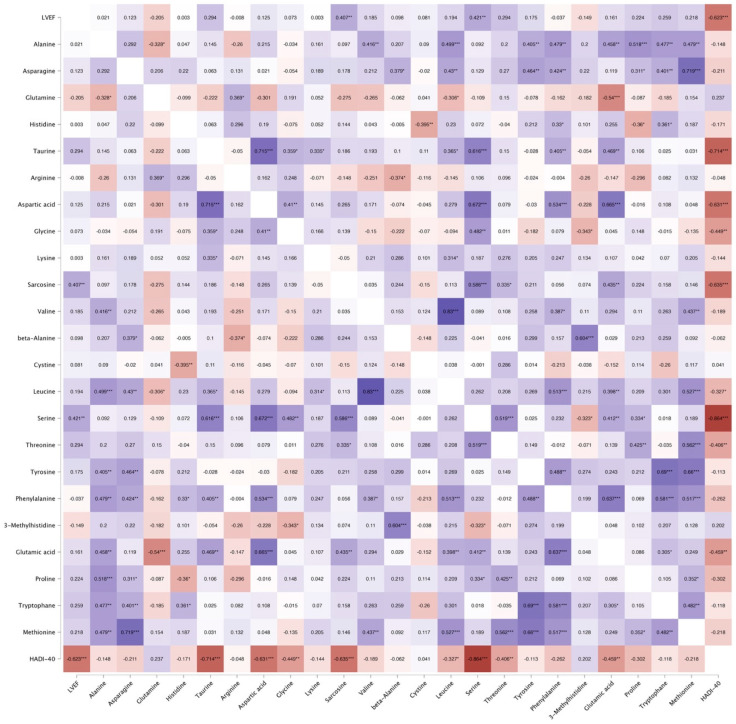
Spearman’s rho heatmap (correlation between LVEF and circulating amino acids). * *p* < 0.05, ** *p* < 0.01, *** *p* < 0.001.

Among individual metabolites, the strongest correlations with LVEF were observed for serine (r = 0.42, *p* = 0.006) and sarcosine (r = 0.41, *p* = 0.008), indicating moderate positive associations between these one-carbon metabolism intermediates and systolic function. In contrast, other amino acids demonstrated weaker or non-significant relationships, reinforcing the absence of a dominant single-metabolite driver. Notably, the composite HASI-40 index showed the strongest overall correlation with LVEF (r = 0.62, *p* < 0.001), supporting the notion that the metabolic phenotype of systolic dysfunction is better captured at an integrative level than by individual analytes as presented in Figure 3.

### 2.6. Uni- and Multivariable Models

The predictive value of HASI-40 for HFrEF was characterized by an odds ratio (OR) of 2.04 (95% CI: 1.33–3.04; *p* < 0.001). The reported odds ratios reflect the increase in odds of HFrEF per one-unit increase in the standardized HASI-40 index.

The receiver operating characteristic (ROC) analysis for LVEF below 40% prediction in relation to HASI-40 yielded an area under the curve (AUC) of 0.862, with a sensitivity of 81.0% and a specificity of 76.2%.

While adjusted in a multivariable model, including age, sex, BMI, diabetes mellitus, dyslipidemia, the HASI-40 was characterized by OR: 5.56 (95% CI: 1.70–18.14, *p* = 0.004); age characterized by OR: 1.32 (95% CI: 1.05–1.65, *p* = 0.017) was found to be predictive of LVEF below 40%.

### 2.7. Receiver Operator Curve Analysis (ROC) for Combined Models

**Figure 4:**
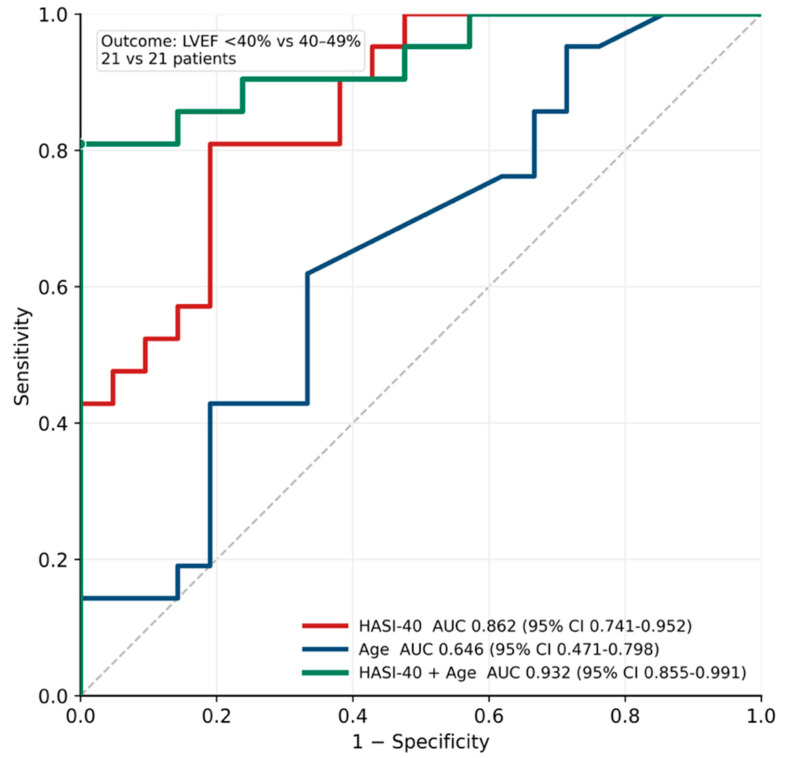
Receiver operating characteristic (ROC) analysis for prediction of LVEF < 40% using HASI-40, age, and the combined HASI-40 + age model.

Receiver operating characteristic (ROC) analysis of LVEF < 40% prediction in relation to HASI-40 (area under the curve (AUC): 0.862, 95% CI: 0.741–0.952), age (AUC: 0.646, 95% CI: 0.471–0.798), and combined AUC of 0.932 (95% CI: 0.855–0.991), with a sensitivity of 90.0% and a specificity of 88.9% (Figure 4).

The ROC analysis assesses HASI-40’s discriminatory performance as a single predictor within the logistic regression framework, whereas the analysis in the present section extends this evaluation by comparing HASI-40 with age and assessing their combined predictive performance. The improvement in AUC observed in the combined model, therefore, reflects the integration of partially independent dimensions of disease severity—metabolic and clinical—rather than a re-estimation of the same model. These analyses are complementary: the former quantifies the association of HASI-40 with HFrEF independent of other variables, while the latter evaluates its performance in a clinically relevant multivariable context.

Although the combined HASI-40 + age model demonstrated improved discrimination compared to either variable alone, formal assessment of incremental predictive value using net reclassification improvement (NRI) or integrated discrimination improvement (IDI) was not performed. The improvement in AUC with age suggests that metabolic and clinical variables capture partially independent dimensions of disease severity. Therefore, the extent to which HASI-40 provides additive clinical utility beyond established risk factors remains to be determined in larger, independently validated cohorts.

### 2.8. Discussion

The present study identifies a metabolic pattern associated with more advanced systolic dysfunction within a heart failure cohort and supports a network-based interpretation of metabolic dysfunction in systolic heart failure. Rather than reflecting isolated perturbations in individual metabolites, the observed pattern is defined by the simultaneous activation of catabolic pathways and depletion of metabolites involved in cytoprotection and one-carbon metabolism. Equal weighting of metabolites simplifies interpretation but assumes equivalent biological contribution, which may not reflect underlying physiology and warrants further investigation using data-driven approaches.

While the concept of metabolic reprogramming in heart failure is well established, the present study contributes to this field by proposing a simplified, biologically interpretable composite index rather than introducing a fundamentally new mechanistic paradigm.

A key observation is that individual amino acids did not retain statistical significance after correction for multiple comparisons, whereas the composite HASI-40 index remained robust. This distinction is not merely statistical but conceptual. It indicates that metabolic dysregulation in HFrEF is inherently multidimensional, and therefore not adequately captured by single-analyte approaches. The observed separation of HASI-40 values around zero reflects cohort-specific standardization and should not be interpreted as a universal diagnostic cutoff. Higher HASI-40 values reflect a relative shift toward proteolytic dominance and reduced metabolic resilience within the studied cohort. The potential value of HASI-40 lies not in replacing echocardiographic classification, but in identifying biologically distinct metabolic phenotypes within the continuum between HFmrEF and HFrEF.

Despite nominal differences observed for methionine and tyrosine [23,24], these metabolites were not included in the HASI-40 index because the selection strategy prioritized representation of distinct, non-overlapping biological domains rather than inclusion of all statistically altered analytes. Specifically, the index was designed to integrate markers of proteolysis (3-methylhistidine), one-carbon metabolism (sarcosine, serine), and cytoprotective buffering (taurine), thereby capturing complementary aspects of systemic metabolic regulation. Methionine and tyrosine, although biologically relevant, were excluded to minimize redundancy within the one-carbon and proteinogenic pathways and to preserve interpretability of the composite construct. This biologically constrained approach was chosen over a purely data-driven selection to reduce the risk of overfitting in a small cohort, but it may have omitted metabolites with additional discriminatory value.

The strength of the HASI-40 index lies in its ability to integrate biologically interconnected processes into a single measurable construct. The HASI-40 index demonstrated a natural separation around zero, with negative values observed predominantly in the LVEF 40–49% group and positive values in the LVEF < 40% group. However, no clinically validated cutoff can be defined from the present dataset, and the index should not be used for individual patient classification.

Our analysis focused on the metabolic distinction between HFmrEF and HFrEF, which likely reflects a shift from compensated metabolic adaptation to overt systemic dysregulation. In patients with HFmrEF, despite measurable impairment in systolic function, key metabolic pathways—particularly those governing mitochondrial energetics, redox balance, and nitrogen handling—appear relatively preserved [25]. This suggests that, at this stage, the organism retains a degree of metabolic flexibility, allowing it to buffer increased energetic demand without substantial perturbation of circulating metabolites. However, our interpretation remains speculative, as direct measures of metabolic flexibility (e.g., exercise testing or dynamic metabolic challenges) were not performed in the present study.

In contrast, HFrEF is characterized by a failure of this adaptive capacity. Sustained neurohormonal activation, impaired tissue perfusion, and chronic inflammatory signaling promote a catabolic milieu, with increased proteolysis and mobilization of amino acids from peripheral compartments [26,27,28]. Crucially, this catabolic drive is not accompanied by adequate compensatory mechanisms. The concomitant reduction in metabolites linked to cytoprotective and one-carbon pathways indicates impaired mitochondrial function and diminished capacity for redox regulation and cellular repair. The resulting metabolic profile is therefore not simply a reflection of increased substrate turnover, but of a broader loss of coordination across interconnected pathways. Within this framework, HFmrEF can be understood as a state in which metabolic reserve is still sufficient to maintain homeostasis. In contrast, HFrEF represents the point at which these reserves are exhausted, giving rise to a distinct and measurable systemic phenotype [29,30].

The key finding is not the alteration of individual metabolites, but the structured nature of the metabolic shift. Lower circulating methionine serum may link cardiac dysfunction to peripheral catabolic processes, particularly skeletal muscle breakdown. This aligns with established observations of cachexia and anabolic resistance in advanced heart failure but extends them by demonstrating that proteolytic activation occurs in parallel with depletion of metabolites involved in cellular resilience. Reduced taurine and serine concentrations suggest impaired cytoprotective buffering, affecting mitochondrial stability, calcium handling, and redox balance—processes that are central to both cardiomyocyte and systemic metabolic function.

The correlation structure shows that no single amino acid dominates association with systolic dysfunction; instead, the strongest relationship is observed with the composite HASI-40 index, supporting the concept that metabolic dysregulation in HFrEF is a coordinated network phenomenon rather than an isolated metabolite effect. The consistency of performance across validation iterations supports the robustness of the composite index despite the modest sample size. The absence of a healthy control group precludes assessment of disease specificity, and it remains unknown whether the observed metabolic profile is unique to heart failure or reflects generalized systemic illness.

Concurrently, reduced serine and sarcosine point to disruption of one-carbon metabolism and methylation flux. These pathways are essential for nucleotide synthesis, epigenetic regulation, and mitochondrial signaling. Their impairment suggests that the observed phenotype reflects not only increased substrate demand but also diminished capacity for metabolic adaptation. These findings support a model in which HFrEF is characterized by a failure of coordinated metabolic responses, whereby adequate regenerative or protective mechanisms do not counterbalance catabolic activation.

From a mechanistic perspective, the index reflects the imbalance between proteolytic activation and metabolic resilience. Elevated 3-methylhistidine suggests increased muscle protein breakdown, consistent with the catabolic state observed in advanced heart failure. Concurrent reductions in sarcosine, serine, and taurine point toward impaired one-carbon metabolism, reduced methylation capacity, and diminished cytoprotective buffering. These pathways are tightly linked to mitochondrial function and redox regulation, suggesting that the observed phenotype reflects a systemic failure of metabolic adaptation.

Importantly, this pattern differentiates HFrEF from HFmrEF at a biological level. Differences in pharmacotherapy, including β-blockers and metformin, were not adjusted for and may influence amino acid metabolism. While HFmrEF is often considered an intermediate clinical category, the relative preservation of metabolites involved in cytoprotection and methylation suggests that metabolic reserve remains partially intact. In contrast, HFrEF appears to represent a threshold beyond which compensatory mechanisms are no longer sufficient, resulting in a distinct and measurable metabolic state.

The improvement in discrimination when HASI-40 is combined with age further supports the concept that metabolic dysregulation and clinical risk capture different dimensions of disease severity. Our study did not demonstrate superiority over data-driven metabolomic classifiers or established clinical models. This reinforces the potential value of integrating metabolomic profiling into clinical assessment frameworks. From a clinical standpoint, the HASI-40 index translates these mechanistic insights into a measurable phenotype. Its robust discrimination of HFrEF and independence from conventional risk factors indicate that it captures disease biology not reflected by age, comorbidity burden, or routine laboratory measures. The improvement in discrimination when combined with age further suggests that metabolic derangement and clinical vulnerability represent complementary dimensions of disease severity rather than redundant information.

In the presented results, while individual metabolites did not remain significant after false discovery rate correction, the composite HASI-40 index retained robust statistical significance, supporting the concept that the metabolic phenotype of HFrEF is better captured at the integrative level than by single analytes. The inclusion of selected metabolites in the HASI-40 index was based on their representation of distinct biological domains rather than statistical significance alone. This approach was chosen to capture complementary aspects of metabolic regulation while minimizing redundancy.

Beyond its diagnostic performance, the HASI-40’s structure has potential implications for patient stratification. By integrating signals of proteolysis and metabolic depletion, the index may identify patients with advanced systemic metabolic impairment, a phenotype associated with frailty, reduced functional reserve, and adverse clinical trajectories. Moreover, the pathways represented—particularly one-carbon metabolism and mitochondrial function—are potentially modifiable, suggesting that metabolic profiling could inform targeted therapeutic strategies.

Recent metabolomic approaches to heart failure classification have increasingly relied on multi-marker panels and data-driven modeling strategies, including machine-learning-based classifiers [31] and heart failure with preserved ejection fraction pathophysiology [32]. Metabolomic analysis pointed out the distinguished profiles of cardiovascular diseases [15,33,34,35] and were investigated as a potential novel marker of risk stratification in heart failure [36,37].

These models often incorporate a large number of metabolites and achieve high discriminatory performance, with reported area under the curve (AUC) values ranging from approximately 0.85 to 0.98 depending on the population and analytical approach. For example, Shah et al. [38] demonstrated that a metabolite panel could improve discrimination of heart failure beyond traditional biomarkers such as natriuretic peptides, while more recent studies using high-dimensional metabolomic data and machine learning algorithms have reported AUC values exceeding 0.90 for heart failure phenotyping and staging. However, these approaches are frequently limited by reduced biological interpretability, model complexity, and a higher risk of overfitting, particularly in smaller cohorts. In contrast, the HASI-40 index was constructed using a biologically guided framework integrating distinct metabolic domains—proteolysis, cytoprotection, and one-carbon metabolism—prioritizing mechanistic interpretability over maximal predictive performance. While the discriminatory ability of HASI-40 (AUC 0.862) is lower than that reported for some data-driven models, its transparent structure may facilitate clinical translation and hypothesis generation. Nevertheless, a direct comparison with existing metabolomic classifiers and a formal evaluation of its incremental predictive value over established clinical models remain necessary to define its relative utility.

From a molecular perspective, the observed metabolic signature likely reflects a convergence of regulatory processes governing amino acid turnover, mitochondrial function, and redox homeostasis rather than isolated pathway perturbations [39,40,41,42]. Increased circulating markers of proteolysis are consistent with activation of ubiquitin–proteasome and autophagy-related pathways, which are known to be upregulated in advanced heart failure and contribute to skeletal muscle catabolism. At the same time, reduced levels of metabolites linked to one-carbon metabolism and cytoprotective buffering suggest impaired flux through pathways essential for methylation reactions, nucleotide synthesis, and antioxidant defense. These processes are tightly coupled to mitochondrial function and cellular stress responses, suggesting that the identified signature may reflect a downstream readout of disrupted cellular metabolic regulation. Importantly, this integrative pattern aligns with emerging concepts of heart failure as a disorder of systemic metabolic coordination, in which failure of adaptive signaling networks—rather than single biochemical defects—drives disease progression. Within this framework, the HASI-40 index should be interpreted not as a surrogate for individual metabolite changes, but as a composite indicator of impaired metabolic network resilience.

From a practical perspective, the potential clinical niche of the HASI-40 index would be within patients already diagnosed with heart failure, particularly those with LVEF in the 40–49% range, where risk of progression toward HFrEF remains uncertain. In this context, the index may serve as a metabolic phenotyping tool rather than a diagnostic test. However, this application remains hypothetical and requires longitudinal validation.

An alternative way to interpret these findings is to consider the HASI-40 index not simply as a biomarker, but as a quantitative reflection of the boundary between metabolic adaptation and metabolic exhaustion in heart failure [43,44]. In earlier stages of systolic dysfunction, metabolic systems retain sufficient redundancy to maintain biochemical homeostasis despite increased energetic demand [45,46]. This buffering capacity, supported by coordinated flux through anabolic, methylation, and cytoprotective pathways, allows the organism to absorb stress without major shifts in circulating metabolites. The transition to HFrEF coincides with the loss of this buffering architecture. Under sustained neurohormonal and inflammatory pressure, catabolic signaling becomes dominant, while pathways responsible for maintaining redox balance and mitochondrial integrity are unable to compensate [47,48]. The magnitude of change does not define the resulting phenotype in any single metabolite, but rather by the collapse of coordination between them. Within this context, HASI-40 can be viewed as a measurable expression of this loss of metabolic resilience—a systems-level inflection point rather than a linear biomarker—offering a framework for understanding heart failure progression as a failure of integrated metabolic control rather than isolated biochemical deficits.

Importantly, the present study did not include a direct comparison of HASI-40 with existing metabolomic classifiers or established clinical risk models for heart failure stratification. As a result, the relative performance and potential superiority of this index over alternative approaches remain uncertain and require evaluation in future studies.

### 2.9. Limitations

Several limitations should be acknowledged. The relatively small sample size and cross-sectional design limit generalizability and preclude causal inference. In addition, the study does not address temporal dynamics or prognostic implications of the identified metabolic phenotype. Nonetheless, the internal consistency of the findings, their biological plausibility, and the composite index’s performance support the validity of the proposed model.

The groups differed significantly in NYHA functional class, indicating more advanced clinical disease in the LVEF < 40% group. This introduces substantial confounding, as metabolic alterations may reflect overall disease severity, nutritional status, or systemic catabolic state rather than systolic dysfunction per se. Therefore, the observed associations cannot be attributed specifically to systolic dysfunction independent of clinical severity. NYHA class was not included in multivariable models, and this limitation should be considered when interpreting the findings. Adjustment for NYHA was not performed because of limited events-per-variable and quasi-separation risk.

Additional limitations include the lack of external validation, which precludes generalizing the HASI-40 index beyond the present cohort. The lack of a healthy control group limits the interpretation of disease specificity. Furthermore, potential confounders such as nutritional status, muscle mass, and pharmacotherapy (e.g., metformin, β-blockers) were not formally adjusted for and may influence circulating amino acid profiles. Moreover, no sensitivity analyses or stratified models were performed to assess the potential influence of specific therapies on amino acid profiles. Therefore, residual confounding related to pharmacological treatment cannot be excluded.

Finally, markers of nutritional status, muscle mass, and protein-energy balance were not assessed, limiting the interpretation of proteolysis-related metabolites such as 3-methylhistidine. In particular, the interpretation of 3-methylhistidine as a marker of proteolysis is limited by the absence of direct measures of skeletal muscle mass, dietary intake, or protein-energy status, which may independently influence circulating levels.

Importantly, NYHA class differed substantially between groups and may represent a dominant driver of metabolic differences. As a result, the HASI-40 index may partly reflect overall clinical severity rather than systolic dysfunction per se.

The HASI-40 index should not be used to guide clinical decision-making in its current form. The index was derived and internally evaluated within a single, small cohort without external validation, defined reference ranges, or assessment of incremental value over established clinical tools. As such, it remains an exploratory, hypothesis-generating measure of metabolic phenotype rather than a clinically actionable biomarker, and its application should be restricted to research settings until further validation is performed.

## 3. Materials and Methods

### 3.1. Study Population

A cross-sectional cohort of adult patients undergoing echocardiographic and metabolic evaluation was analyzed. Patients were stratified into HFrEF (LVEF < 40%) and the reference group (LVEF 40–49%). Clinical covariates included age, sex, and BMI. The exclusion criteria included acute heart failure decompensation requiring hospitalization within the last six months, leading to hospitalization, followed by food allergies or any dietary restrictions that could impair amino acid profiling.

### 3.2. Clinical Evaluation

Blood samples were collected in the morning after overnight fasting to minimize circadian variation. The laboratory testing was followed by echocardiography performed by an experienced echocardiographer. All patients underwent coronary angiography in the tertiary hemodynamic center.

### 3.3. Amino Acids/Metabolomic Profiling

Peripheral blood samples for amino acid profiling were obtained on admission. LC quantified the concentration of amino acids in the serum–MS/MS using the MassChrom Amino Acid Analysis kit (Chromsystems, Gräfelfing, Germany). 25 µL of serum was mixed with 50 µL of internal standard and 400 µL of precipitation reagent, vortexed, and centrifuged at 16,000× *g* for 5 min. The supernatant was analyzed on a 5500+ QTRAP mass spectrometer (Sciex, Framingham, MA, USA) coupled with an Agilent 1260 Infinity II HPLC system. Chromatographic separation was performed on the manufacturer’s analytical column using the recommended binary gradient. Data acquisition and quantification were done in Analyst (v1.7.3) and SciexOS (v3.3). Calibration and two-level QC samples were included in each batch. All analytes met QC acceptance criteria. The intra-assay CV was 4.98%. Internal validation was performed using repeated 5-fold cross-validation (100 repetitions), and performance metrics were averaged across iterations.

Raw metabolomic data were processed using manufacturer software with internal standard normalization. Quality control samples were used to ensure analytical stability. No additional batch correction or imputation procedures were applied. Peak integration and quantification were performed using manufacturer-provided algorithms, and no additional normalization beyond internal standards was applied.

Raw signal intensities were processed using the manufacturer’s software with internal standard-based normalization, followed by concentration calculation using calibration curves. No dimensionality reduction, feature selection, or pathway enrichment algorithms were applied, as the analysis was restricted to a predefined panel of quantified amino acids. Thus, the metabolomic analysis represents a targeted, hypothesis-driven approach rather than an untargeted or systems-level omics workflow.

Amino acids were analyzed in relation to the functional role, as follows:

1. Proteinogenic Amino Acids—directly incorporated into proteins; reflect nutritional status, protein turnover, and anabolic balance. In HFrEF, they reflect global protein turnover, anabolic resistance, and substrate availability. The proteinogenic amino acids include essential (histidine, isoleucine, leucine, lysine, methionine, phenylalanine, threonine, tryptophan, and valine) and non-essential/conditionally essential (alanine, arginine, asparagine, aspartate, cysteine, glutamate, glutamine, glycine, proline, serine, and tyrosine).
2. Methylation Pathway Intermediates, which are linked to one-carbon metabolism, epigenetics, mitochondrial function, and redox balance. Their role in HFrEF is linked with impaired methylation flux, disrupted one-carbon metabolism, mitochondrial dysfunction, and oxidative stress. This group includes the following amino acids: sarcosine, glycine, serine, methionine.
3. Muscle Catabolism Markers that reflect proteolysis, cachexia, and systemic catabolic activation, including core markers such as 3-Methylhistidine (3-MH), followed by alanine and glutamine.

### 3.4. Biomarker Derivation

We proposed the Heart Failure Amino Acid-Derived Systolic Index-40 (HASI-40) index that integrates metabolites spanning three biologically distinct domains—muscle proteolysis, methylation and one-carbon metabolism, and cytoprotective buffering—thereby capturing a multidimensional metabolic failure state in HFrEF. The HASI-40 index is a relative, dimensionless measure reflecting within-cohort metabolic variation and should not be interpreted as an absolute biomarker or diagnostic threshold.

The HASI-40 index (Heart Failure Amino Acid-Derived Systolic index-40) was defined as:

HASI-40 = z(3-Methylhistidine1) − z(Sarcosine) − z(Taurine) − z(Serine)

Standardization was performed using z-scores, defined as: z = (individual value − cohort mean)/standard deviation. All z-scores were calculated across the entire study cohort.

Alternative combinations of metabolites were not systematically screened or optimized using data-driven approaches. The final composition of the index was defined a priori based on biological rationale rather than comparative testing of multiple candidate models.

The HASI-40 index was constructed based on predefined biological directionality rather than data-driven coefficient optimization. Specifically, metabolites reflecting proteolysis were assigned positive contributions, while metabolites associated with cytoprotection and one-carbon metabolism were assigned negative contributions. This approach was chosen to preserve mechanistic interpretability and reduce the risk of overfitting. Biological rationale and limitations of components included in the HASI-40 index are presented in Table 4.

Importantly, inclusion of metabolites in the HASI-40 index was independent of statistical significance after multiple-testing correction and was based exclusively on predefined biological roles.

As presented, the HASI-40 index integrates metabolites from complementary biological domains rather than selecting analytes solely on the basis of statistical significance, prioritizing mechanistic interpretability over data-driven optimization.

Although several metabolites demonstrated nominal differences between groups, only those representing distinct biological domains (proteolysis, one-carbon metabolism, and cytoprotection) were included to avoid redundancy and multicollinearity in the HASI-40 index. Equal weighting was applied due to the absence of prior evidence supporting differential contribution and to preserve interpretability. However, this approach is inherently simplified and does not account for potential nonlinear or weighted relationships, which should be explored in future data-driven models.

No sensitivity analyses were performed to evaluate the impact of alternative weighting schemes or nonlinear relationships between components. Consequently, the assumption of equal contribution of each metabolite should be interpreted as a simplifying approximation rather than a biologically validated model.

### 3.5. Statistical Analysis

Statistical analyses were performed using GraphPad Prism version 10.2.3 (GraphPad Software, Boston, MA, USA). Continuous variables were first assessed for distributional characteristics and, given the small sample size and non-Gaussian distributions of several variables, are presented as medians with interquartile ranges (Q1–Q3). Categorical variables are presented as counts and percentages.

Covariates were selected based on clinical relevance and data availability rather than formal model selection procedures.

Comparisons between the two study groups (LVEF 40–49% and LVEF < 40%) were performed using non-parametric or exact methods as appropriate. Continuous variables were compared using the Mann–Whitney U test, whereas categorical variables were compared using Fisher’s exact test or chi-square test, depending on expected cell counts.

For metabolomic profiling, individual amino acid concentrations were compared between groups using the Mann–Whitney U test. Because multiple metabolites were tested simultaneously, raw *p*-values were adjusted using the Benjamini–Hochberg false discovery rate (FDR) procedure to reduce the risk of type I error arising from multiple comparisons. Both raw *p*-values and FDR-adjusted q-values are reported.

To explore associations between circulating amino acids and left ventricular ejection fraction, Spearman’s rank correlation coefficient (rho) was used. This approach was selected because it does not assume normality and is robust to monotonic relationships in relatively small datasets. Correlation strength was interpreted descriptively, and corresponding *p*-values were reported.

The HASI-40 index was constructed from standardized metabolite values (z-scores) to place metabolites measured on different concentration scales into a common metric. The index was defined a priori according to biological directionality as:

HASI-40 = z(3-methylhistidine) − z(sarcosine) − z(taurine) − z(serine)

Metabolites interpreted as reflecting proteolytic activation were assigned a positive contribution, whereas metabolites linked to cytoprotection and one-carbon metabolism were assigned negative contributions. This biologically guided approach was chosen to preserve mechanistic interpretability and reduce the risk of overfitting associated with purely data-driven weighting.

To evaluate the association between HASI-40 and the presence of HFrEF (LVEF < 40%), logistic regression analysis was performed. Odds ratios for HASI-40 represent the change in odds of HFrEF per 1-unit increase in the standardized index.

First, univariable models were calculated. Subsequently, a multivariable logistic regression model was constructed, including clinically relevant covariates selected a priori, namely age, sex, body mass index, diabetes mellitus, and dyslipidemia. Results are presented as odds ratios (ORs) with 95% confidence intervals (95% CIs).

The discriminatory performance of HASI-40 for identifying HFrEF was assessed using receiver operating characteristic (ROC) curve analysis. The area under the ROC curve (AUC) with 95% confidence intervals was calculated to quantify classification performance. Sensitivity and specificity were derived from the optimal cutoff identified within the ROC framework. In addition to HASI-40 alone, ROC analysis was performed for age and for the combined HASI-40 + age model to determine whether integrating metabolic and clinical information improved discrimination. No comparison with alternative metabolomic or clinical indices was performed; therefore, the relative performance of HASI-40 remains uncertain.

To reduce optimism bias and assess the robustness of model performance, internal validation was performed using repeated cross-validation in GraphPad Prism. The consistency of the discriminatory performance across validation iterations provided supportive evidence of model stability despite the modest sample size.

All tests were two-sided, and a *p*-value < 0.05 was considered statistically significant unless otherwise specified. For metabolite panel analyses, emphasis was placed on FDR-adjusted results when interpreting individual analytes, whereas the composite HASI-40 index was interpreted as an integrated biologically informed measure of metabolic dysregulation.

## 4. Conclusions

HASI-40 index reflects coordinated metabolic alterations associated with more advanced systolic dysfunction within a heart failure cohort. However, due to methodological limitations—including a small sample size, the absence of external validation, and a lack of disease-specificity assessment—it should be considered an exploratory research tool rather than a clinically applicable biomarker.

These findings support the concept that systolic dysfunction is associated with coordinated metabolic alterations, characterized by disruptions in proteolysis, cytoprotection, and methylation pathways. The HASI-40 index should be considered an exploratory tool requiring external validation before clinical application.

The proposed index operationalizes this concept and provides a biologically interpretable research framework that may inform future development of clinically applicable tools, pending external validation. At present, no specific clinical action can be recommended based solely on the HASI-40 score. Its potential role may lie in research settings for phenotyping metabolic dysfunction or in future risk stratification models, pending external validation and demonstration of incremental prognostic value.

## Figures and Tables

**Table 1:** Groups characterization.

Parameters	LVEF 40–49% Group n = 21	LVEF < 40 Group n = 21	*p*
Demographical:			
Age (years) (median (Q1–Q3))	66 (50–69)	68 (63–74)	0.107
Sex (male (%))	12 (57)	14 (67)	0.540
BMI (kg/m^2^) (median (Q1–Q3))	28.3 (27.2–31.1)	26.9 (25.0–30.9)	0.191
Obesity (BMI > 30 kg/ms) (n (%))	8 (38)	6 (29)	0.528
Clinical status (NYHA classification):			
I/II (n (%))	12 (57)	3 (14)	0.009
II (n (%))	9 (43)	10 (48)	1.000
II/III (n (%))	0 (0)	1 (5)	1.000
III (n (%))	0 (0)	7 (33)	0.008
Co-morbidities:			
Arterial hypertension (n (%))	15 (71)	13 (62)	0.506
Diabetes mellitus type 2 (n (%))	4 (19)	7 (33)	0.302
Dyslipidemia (n (%))	20 (95)	17 (81)	0.343
COPD (n (%))	2 (10)	1 (5)	0.511
Kidney dysfunction * (n (%))	6 (29)	5 (24)	0.740
Family CVD history (n (%))	3 (13)	6 (29)	0.137
Therapy:			
B-blockers (n (%))	13 (62)	8 (38)	0.131
ACE-I (n (%))	9 (43)	6 (29)	0.348
ARB (n (%))	4 (19)	2 (10)	0.395
Statins (n (%))	15 (71)	13 (62)	0.528
Metformin (n (%))	4 (19)	7 (33)	0.302
SGLT2-i (n (%))	7 (33)	7 (33)	1.000
Loop diuretics (n (%))	12 (57)	14 (67)	0.760
MRA (n (%))	12 (57)	14 (67)	0.760

Abbreviations: ARB—angiotensin receptor blocker, ACE-I, angiotensin converting enzyme inhibitor, BMI—body mass index, COPD—chronic obstructive pulmonary disease, CVD—cardiovascular disease, kg—kilogram, MRA—mineralocorticoid receptor antagonist. m^2^—square meter, n—number, NYHA—New York Heart Association, SGLT2-i—sodium-glucose co-transporter inhibitor 2, Q—quartile, * GFR below 60 mL/kg/min.

**Table 2:** Laboratory, echocardiographic, and angiographic results in the analyzed groups.

Parameters	LVEF 40–49% Group n = 21	LVEF < 40% Group n = 21	*p*
Laboratory results			
Hematocrit (median (Q1–Q3))	43 (40–45)	44 (41–45)	0.293
Glomerular filtration rate (GFR) (mL/kg/min) (median (Q1–Q3))	73 (64–81)	70 (63–74)	0.531
ALT (IU/L) (median (Q1–Q3))	40 (24–55)	26 (21–38)	0.106
BNP (pg/mL) (median (Q1–Q3))	402 (304–470)	787 (337–978)	0.222
Uric acid (μmol/L)(median (Q1–Q3))	329 (294–379)	338 (217–363)	0.661
Lipoprotein (a) (mg/dL) (median (Q1–Q3))	2.4 (2.1–2.9)	2.6 (1.4–4.8)	0.915
Total cholesterol (mmol/L) (median (Q1–Q3))	4.8 (3.6–5.7)	4.3 (3.3–5.6)	0.871
Echocardiography			
Transthoracic echocardiography results:			
LVEDD (mm) (median (Q1–Q3))	50 (45–52)	54 (50–57)	0.053
IVS (mm) (median (Q1–Q3))	11 (10–12)	10 (9–12)	0.447
LA (mm) (median (Q1–Q3))	37 (35–41)	42 (39–46)	0.065
MItral insufficiency moderate (n (%))	2 (10)	4 (19)	0.395
LVEF (mm) (median (Q1–Q3))	45 (42–46)	35 (30–38)	<0.001
Coronary angiography results			
Coronary angiography proven significant disease:			
1—vessel disease (n (%))	4 (19)	4 (19)	1.000
2—vessel disease (n (%))	1 (5)	3 (14)	0.311
3—vessel disease (n (%))	5 (24)	1 (5)	0.085

Abbreviations: ALT—alanine aminotransferase, BNP—brain natriuretic peptide, IVs—interventricular septum, kg—kilogram, LA—left atrium, LVEDD—left ventricular end-diastolic diameter, LVEF—left ventricular ejection fraction, min—minutes, mm—millimole, mL—milliliter, n—number, Q—quartile.

**Table 3:** Amino acid concentrations (μmol/L) according to LVEF category with raw *p*-values and FDR-adjusted q-values (Benjamini–Hochberg).

Amino Acids (μmol/L) (Median (Q1–Q3))	LVEF 40–49% Group n = 21	LVEF < 40% Group n = 21	*p*	FDR q
Proteinogenic Amino Acids:				
Essential:				
Histidine	95.6 (87.9–102.6)	90.7 (81.5–109.8)	0.435	0.639
Isoleucine	61.6 (57.4–68.8)	58.3 (52.8–62.4)	0.724	0.839
Leucine	145 (136–161)	141 (119–150)	0.132	0.412
Lysine	202 (161–217)	199 (172–228)	0.715	0.839
Phenylalanine	74.8 (68.9–87.3)	73.2 (61.6–88.0)	0.132	0.412
Methionine	26.8 (23.6–28.2)	25.3 (19.7–27.1)	0.035 *	0.268
Threonine	117 (99–140)	107 (95–123)	0.187	0.422
Tryptophan	64.3 (59.0–68.3)	53.0 (45.8–61.3)	0.061	0.268
Valine	253 (239–297)	246 (230–264)	0.220	0.422
Non-essential/conditionally essential:				
Alanine	417 (376–443)	412 (352–465)	0.687	0.839
Arginine	83.7 (76.5–97.1)	83.2 (74.3–97.5)	0.980	0.980
Asparagine	47.8 (45.0–52.2)	46.1 (42.1–50.4)	0.320	0.513
Aspartic acid	25.6 (20.6–35.3)	20.5 (17.1–29.0)	0.187	0.422
Cystine	56.4 (49.1–65.7)	56.2 (50.9–66.8)	0.980	0.980
Glutamine	541 (503–595)	612 (572–661)	0.131	0.412
Glycine	250 (209–275)	230 (207–281)	0.881	0.968
Proline	192 (171–221)	179 (168–196)	0.222	0.422
Tyrosine	70.7 (65.5–84.1)	62.3 (53.9–75.9)	0.032 *	0.238
One-carbon metabolism:				
Sarcosine	1.7 (1.4–2.1)	1.2 (1.0–1.5)	0.004 *	0.080
Glycine	250 (209–275)	230 (207–281)	0.881	0.968
Serine	147 (130–161)	123 (108–146)	0.012 *	0.135
Methionine	26.8 (23.6–28.2)	25.3 (19.7–27.1)	0.053	0.268
Taurine	161 (143–176)	116 (99–152)	0.018 *	0.120
Muscle catabolism:				
3-Methylhistidine (3-MH)	12.2 (7.0–22.2)	14.1 (7.9–29.1)	0.725	0.839
Beta-Alanine	3.0 (2.5–3.9)	2.9 (2.4–3.5)	0.614	0.839
Glutamine	541 (503–595)	612 (572–661)	0.131	0.412
HASI-40 index (median (Q1–Q3))	−2.025 (−3.021–−0.429)	1.890 (0.642–3.166)	<0.001	0.011

Abbreviations: L—liter, LVEF—left ventricular ejection fraction, n—number, μmol—micromole, Q—quartile, * statistically significant.

**Table 4:** Biological rationale and limitations of components included in the HASI-40 index.

Metabolite	Biological Domain	Direction in Index	Reason for Inclusion	Limitation
3-Methylhistidine (3-MH)	Muscle proteolysis/catabolic activity	Positive (+)	Established marker of myofibrillar protein breakdown; reflects systemic catabolic activation observed in advanced heart failure	Not specific to heart failure; influenced by muscle mass, diet (meat intake), and protein-energy malnutrition
Sarcosine	One-carbon metabolism/methylation flux	Negative (−)	Intermediate of glycine–serine metabolism; reflects methylation capacity and mitochondrial function	Levels may be influenced by renal function and folate metabolism; not specific to cardiac pathology
Serine	One-carbon metabolism/cytoprotective metabolism	Negative (−)	Central substrate for nucleotide synthesis, redox balance, and mitochondrial pathways; reduced levels suggest impaired metabolic resilience	Broad metabolic role reduces specificity; influenced by nutritional status and systemic illness
Taurine	Cytoprotective buffering/mitochondrial stability	Negative (−)	Involved in calcium handling, antioxidant defense, and membrane stabilization; depletion linked to impaired cellular protection	Affected by diet, renal handling, and comorbidities; not unique to heart failure

## Data Availability

Data supporting the reported results are available upon reasonable request from the corresponding authors via e-mail.
